# Sepiolite-Based Nanogenerator Driven by Water Evaporation

**DOI:** 10.3390/nano15130983

**Published:** 2025-06-25

**Authors:** Liwei Zhao, Guoxing Jiang, Xing Zhang, Chunchang Wang

**Affiliations:** Laboratory of Dielectric Functional Materials, School of Materials Science & Engineering, Anhui University, Hefei 230601, China; ms22301080@ahu.edu.cn (L.Z.); ms23201029@ahu.edu.cn (G.J.); ms22201002@ahu.edu.cn (X.Z.)

**Keywords:** sepiolite, electricity generation, energy harvesting, evaporation, hydrovoltaic effect

## Abstract

This work introduces a new type of water evaporation-driven nanogenerator (S-WEG) utilizing the natural mineral sepiolite, which capitalizes on its hierarchical nanoporous architecture and intrinsic hydrophilicity to harvest energy from ambient humidity through capillary-driven evaporation. The S-WEG, fabricated via a facile drop-coating drying method, demonstrates remarkable mechanical flexibility and sustained operational reliability. Our results demonstrate that by optimizing evaporation height and width, the S-WEG can generate a short-circuit current of ~0.6 μA and an open-circuit voltage of ~0.9 V. Through series and parallel configurations of multiple S-WEG units, the current and voltage outputs can be effectively amplified to power small-scale electronics.

## 1. Introduction

Amid growing global energy crises and environmental pollution, the reliance on fossil fuels has caused severe consequences. Developing renewable and clean energy is now imperative, and extracting energy from the environment offers a viable solution to these challenges [1]. In recent years, numerous ingenious power generators have been designed. Examples include piezoelectric nanogenerators [2,3,4], triboelectric nanogenerators [5,6,7], moisture–electric generators [8,9,10], and water evaporation-induced generators (WEGs) [11,12,13]. Among them, WEGs stand out because of their simple structure, ease of preparation, and ability to spontaneously and continuously harvest energy across a wide range of environmental humidity levels. WEGs utilize water as a medium to convert ambient environmental energy into electricity and generate direct current (DC) output. As a novel green energy technology, WEGs have attracted widespread attention [14].

In 2017, Guo et al. demonstrated that water evaporating from the surface of various nanostructured carbon materials could be used for electricity generation. They found that the evaporation of centimeter-sized sheets of carbon black could reliably produce sustained voltages of up to 1 V under ambient conditions [15]. Since then, researchers have made considerable efforts to improve the energy conversion efficiency of WEGs by optimizing the material composition, modifying the material surface, and rationally designing the device configuration [16,17,18]. Cheng et al. developed an evaporation-driven power generation system based on MOF nanochannels, employing MOF-801 with high porosity, charged surfaces, and hydrophilicity to enhance the output performance of evaporation-induced electricity generation [19]. In addition, some natural materials, such as fabrics and wood, can also produce evaporation-driven electricity. For example, Sun et al. provided novel insights for optimizing efficient evaporation-powered generation systems by functionally coupling evaporation interfaces through oxygen-containing functional group (OCG) gradients, based on the abundance of reactive OCGs in wood-derived materials. This approach facilitates effective energy utilization in natural environments [20]. In terms of material selection, the main materials currently available are carbon [21], solid oxides [22], microorganisms [23], and polymers [24]. Carbon-based materials, such as carbon black [25,26,27,28], carbon nanotubes [29,30,31,32], graphene [33,34,35,36], etc., are widely used for WEG fabrication because of their good conductivity and large surface area. However, challenges such as the complexity of the fabrication process and the high cost of carbon-based materials have limited their large-scale applications [37]. Therefore, it is necessary to select more suitable materials by elucidating the underlying mechanisms and choosing more appropriate alternatives.

**Figure 1:**
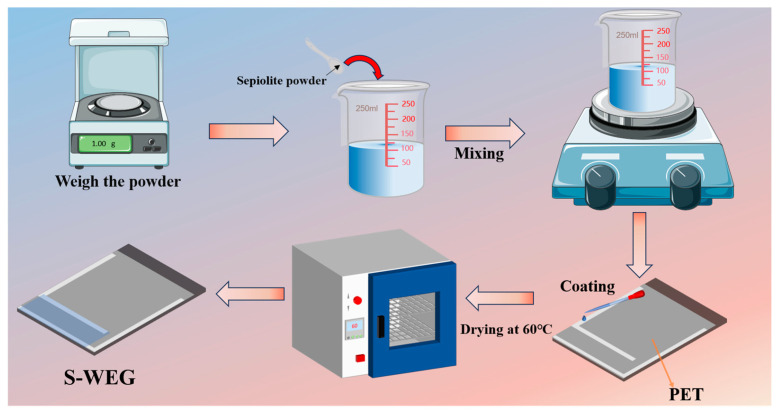
Schematic diagram of the S-WEG fabrication process.

Sepiolite is a naturally occurring, non-toxic, odorless, asbestos-free, and non-radioactive fibrous hydrated magnesium silicate clay mineral. Based on the mechanism of the hydrovolt effect, the flow rate of water, the pore size of the nanochannels, the zeta potential of the material surface, and the charge collection efficiency are a few key factors that affect the output power density. Compared with commonly used porous ceramic materials, sepiolite has a higher specific surface area than most porous materials. In addition, because sepiolite has naturally arranged nanochannels, its water flux is more than twice that of Al_2_O_3_, which makes sepiolite have better power generation potential in water evaporation nanogenerators. Compared with carbon black materials, sepiolite has hydrophilicity and unique nanochannels, while carbon black materials usually need to be modified to have hydrophilicity. Sepiolite also has a higher specific surface area, richer surface functional groups, and wider natural reserves than carbon black materials. In addition, sepiolite and inorganic ceramics usually have good thermal stability and chemical stability [38]. The following merits make sepiolite a highly promising WEG material. Firstly, sepiolite demonstrates exceptional stability, maintaining its structural integrity at temperatures up to 350 °C with a remarkable heat resistance range of 1500–1700 °C, making it suitable for high-temperature applications. Secondly, its unique structure endows it with zeolite-like properties, featuring an extensive network of water channels and pores coupled with an extraordinarily large specific surface area for substantial water adsorption. Finally, sepiolite is cost-effective and readily available. Based on these superior properties, we selected sepiolite as the water-responsive material for fabricating a sepiolite-based hydrovoltaic generator (S-WEG, Figure 1). We, herein, systematically investigate the effects of evaporation height, width, and various environmental conditions on the performance of the S-WEG. The experimental results demonstrate that the optimized S-WEG device achieves peak performance metrics of 0.6 μA short-circuit current and 0.9 V open-circuit voltage.

## 2. Experiment

### 2.1. Materials

Aluminum foil and polyethylene terephthalate (PET) substrate were obtained commercially. Sepiolite powder (SEP) was purchased from Shanlin Stone Language Flagship Store (Xiangxi, China).

### 2.2. S-WEG Fabrication

The S-WEG was prepared by a simple drop-coating drying method, as shown in Figure 1. First, 1 g of sepiolite was weighed and added to 65 mL of deionized water; then, the mixture was stirred in a magnetic stirrer for 1 h. Next, the sepiolite solution was dropped onto a PET substrate with attached aluminum electrodes, using PET to carry sepiolite and affix the PET to an acrylic board to prevent curling. After drying in an oven at 60 °C, the S-WEG was obtained.

### 2.3. Characterizations

The crystal structure was analyzed using an X-ray diffractometer (XRD, Rigaku Smartlab Beijing Co., Beijing, China). The sample surface was observed by a scanning electron microscope (SEM) with the SEM Regulus 8230 (Hitachi, Tokyo, Japan). The infrared spectra (FT-IR) were measured using an FTIR microscope from Bruker, Ettlingen, Germany. The water contact angle was measured by the contact angle measuring instrument DSA30S (KRUSS, Hamburg, Germany). A multimeter was used to ensure that no short circuit occurred after the test. The output voltage and current of the S-WEG were measured by a Keithley 6517B electrometer (Johnston, OH, USA).

## 3. Results and Discussion

### 3.1. Characterization of the S-WEG

**Figure 2:**
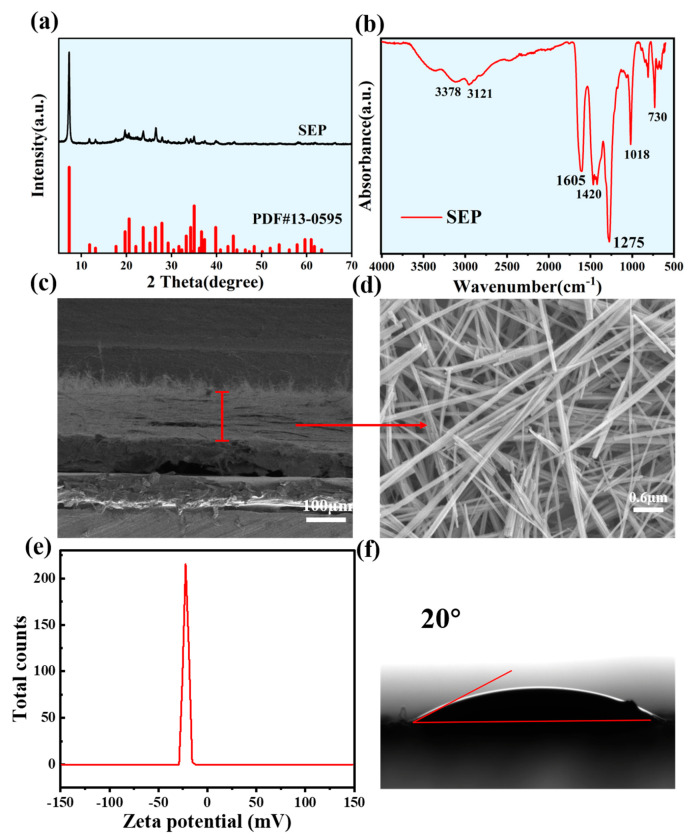
(**a**) XRD pattern of the sepiolite membrane. (**b**) Infrared spectrum of sepiolite. (**c**,**d**) Cross-section SEM images of the sepiolite film. (**e**) Zeta potential of sepiolite. (**f**) Water contact angle of the sepiolite film.

As shown in Figure S1, the structure of sepiolite is composed of two layers of silicon–oxygen tetrahedral sheets and one layer of magnesium–oxygen octahedral sheets, forming a continuous chain-like layered structure, belonging to the orthorhombic system, and the space group is Pncn [39]. Generally, the unit cell parameters of sepiolite are *a* = 4.96 Å, *b* = 15.42 Å, and *c* = 10.77 Å. According to the standard PDF card No. 13-0595 for sepiolite, the XRD pattern of the sepiolite membrane, as shown in Figure 2a, indicates that the characteristic peak at 7.4° corresponds to the (110) crystal plane of sepiolite. The diffraction peaks of quartz, talc, dolomite, and calcite are almost absent, which proves the high purity of our sepiolite. The XRD peaks are sharp, narrow, and symmetrical, with higher peaks for the main elements, indicating that the composition of sepiolite has good crystallinity and large grain size. Sepiolite has a unique structural arrangement, such as the layered talc structure. Its cavity (structural tunnel) is aligned with the *c*-axis and extends along the *b*-axis direction. These cavities constitute nano-sized pores and form unique nanochannels. The formation of nanochannels is beneficial for the increase in the water transport rate. In order to further clarify its composition, an FTIR measurement was carried out (Figure 2b). The result shows that the peaks at ~1275 and 1018 cm^−1^ are related to the vibration of Si-O. The peak at 730 cm^−1^ is related to the Si-O-Si symmetric stretching vibration. Two broad absorption bands at 3378 and 3121 cm^−1^ in the infrared spectrum indicate the stretching vibration of H_2_O in sepiolite. In the absorbance spectrum, the stretching vibration mode of H₂O in sepiolite appears predominantly within the range of 3400–3000 cm^−1^, which is consistent with the literature [40]. The peak at 1605 cm^−1^ is attributed to δ(H_2_O). Figure 2c,d show the SEM images of the S-WEG power generation layer. The cross-sectional SEM image shows that the thickness of the sepiolite film above the PET substrate is about ~160 μm. The sepiolite is evenly spread on the PET substrate, and there are many large-sized nanochannels between the sepiolite films, as shown in Figure 2d. These nanochannels are conducive to promoting the flow and transport of water in the membrane. At the same time, it is well known that sepiolite is usually negatively charged. To verify this, we measured the zeta potential of sepiolite, as shown in Figure 2e. We determined that the zeta potential of sepiolite in water was −22.3 mV. Therefore, sepiolite-based water evaporation-driven nanogenerators are also negatively charged. In addition, the S-WEG is expected to exhibit hydrophilicity. In this study, a solid dispersion system was employed to prepare samples for zeta potential measurements. The zeta potential of the commercial sepiolite powder was determined using a nanoparticle size analyzer with a laser diffraction technique, where the powder was dispersed in deionized water at a pH of 7. When an electrolyte solution flows through the nanochannel of sepiolite material driven by water evaporation, the charged channel wall attracts counter ions and forms an electric double layer (EDL). When the liquid flows, the dynamic change in the EDL will produce an electrodynamic phenomenon. When the electrolyte is driven by a pressure gradient through a channel with a size equal to the Debye length of the solution, the charged wall in the narrow channel will cause the EDL to overlap. As the electrolyte continues to flow, the ion concentration varies in the channel, so that the potential difference can be detected, resulting in a current potential. The directional movement of this single-charged ion in the channel generates a flow current. As shown in Figure 2f, when a water droplet was placed on the surface of the sepiolite film, the contact angle between the sepiolite film and water reached 20° within 8 s, demonstrating its excellent hydrophilic properties. Hydrophilicity affects capillary action and can synergistically enhance the electric double layer effect with zeta potential. These results indicate that sepiolite is a porous hydrophilic material with promising potential for water evaporation-induced power generation.

### 3.2. Output Performance and Principle of the S-WEG

In order to study the power generation performance of the S-WEG, we put the S-WEG into a beaker and added deionized water until the bottom electrode was completely immersed. Then, the upper electrode of the S-WEG was connected to the positive electrode of the electrometer with a crocodile clip, and the lower electrode of the S-WEG was connected to the negative electrode of the electrometer with a crocodile clip, in order to measure the open-circuit voltage (V_oc_) and short-circuit current (I_sc_) of the device.

**Figure 3:**
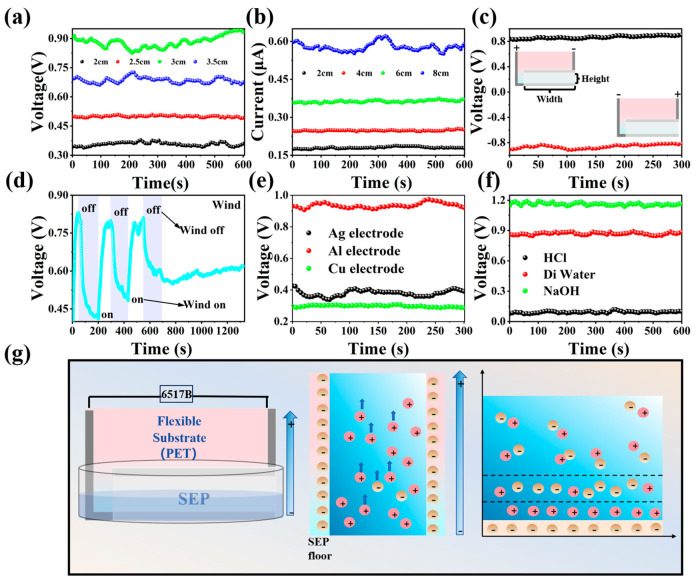
(**a**) The output voltage and current of the S-WEG under different film heights and (**b**) widths. (**c**) Change in the connection between the S-WEG and 6517B to produce opposite polarity equal voltage. Effects of (**d**) wind, (**e**) electrode, and (**f**) electrolyte on device performance. (**g**) Schematic diagrams of water evaporation power generation.

When the deionized water is transported upward along the unique nanochannel inside the sepiolite and contacts the upper electrode over time through capillary action and evaporation, an electrical signal will be generated. With an increase in time, the contact area between water and the upper electrode increases. When the evaporation rate of sepiolite on the upper electrode and the upward transport rate of water through capillary action reach a dynamic equilibrium, a stable humidity difference is obtained between the upper and lower electrodes, and the signal tends to be stable. In order to determine the optimal device size of the device, we used control variables to study the factors affecting the performance of the S-WEG. Firstly, we studied the effects of evaporation height and evaporation width on the power generation performance of the S-WEG. When the width of the power generation layer was fixed at 8 cm, we changed the evaporation height of the device. As shown in Figure 3a, when the evaporation height increases from 2 to 3 cm, the V_oc_ of the S-WEG increases from 0.37 to 0.90 V. When the evaporation height further increases to 3.5 cm, the output V_oc_ shows a downward trend. This phenomenon is due to the fact that as the evaporation height increases, the device can be regarded as a series of batteries connected in series. The more batteries that are connected in series, the higher the evaporation height, and the output performance of the device also improves. When it increases to a certain height, the resistance of water also increases, resulting in a decrease in the output signal. Then, we controlled the evaporation height at 3 cm to explore the effect of the evaporation width on the output performance of the device. As shown in Figure 3b, when the width increases from 2.0 to 8 cm, the output I_sc_ increases from 0.17 to 0.6 μA. It can be seen that the output signal increases with the increase in the width. Under this condition, the output signal is positively correlated with the width. This phenomenon can be attributed to the fact that in the horizontal direction, the device can be viewed as parallelly connected batteries, and the output signal increases as the evaporation width increases. Therefore, when the power generation layer width of the device is 8 cm and the height is 3 cm, the S-WEG can achieve the best performance output of V_oc_ = 0.9 V and I_sc_ = 0.6 μA. Except for special cases, the size of the S-WEG was controlled at 3 × 8 cm^2^ in the subsequent experiments.

In order to verify that the electrical output signal is mainly caused by the humidity difference caused by moisture evaporation, and to exclude the primary battery effect, we switched the upper and lower electrodes of the device to the electrometer when the output signal was stable. The opposite connection modes, as shown in Figure 3c, had similar voltage outputs but opposite voltage polarities. This fact indicates that the direction of the current is determined by the direction of water evaporation, eliminating the influence of the primary battery effect. As shown in Figure S2, the device was placed in a beaker and sealed with a cling film. After sealing, the humidity increases slowly, which inhibits the evaporation rate of water. The output performance of the device drops sharply. As shown in Figure S3, when the device is covered by an acrylic box, the humidity in the box is increased by a humidifier. It can be seen that as the humidity increases, the output performance of the device decreases. In order to further determine if the output performance is caused by water evaporation, we placed a fan about 20 cm away from the device. When airflow is introduced by the fan, the voltage generated through water evaporation increases. Upon cessation of the airflow, the voltage gradually returns to its baseline value, as illustrated in Figure 3d. The above experiments show that the output performance is closely related to water evaporation. Figure 3e shows the effect of the electrode on device performance. The voltage drop obtained using silver paste as the electrode is ~0.3 V, while the voltage drop obtained using copper foil as the electrode is ~0.4 V. This is because when using an aluminum electrode, aluminum absorbs the positive charge of water, making the water rich inOH-. OH- reacts with sepiolite, causing more ions in the lower electrode and a higher concentration difference between the upper and lower electrodes, resulting in a higher output signal. In Figure 3f, it can be seen that when using a HCl solution instead of deionized water for water evaporation, the generated voltage is close to 0 V. When using a NaOH solution, a voltage of approximately 1.2 V is generated. In addition, as shown in Figure S4, the output voltage of the S-WEG can be stably maintained at about 0.9 V for more than 5000 s, indicating that the S-WEG has excellent stability.

Figure 3g schematically illustrates the mechanism of water evaporation power generation. The upper electrode is connected to the positive electrode of an electrometer, and the lower electrode is connected to the negative electrode of the electrometer. The lower electrode is completely covered by deionized water, and the upper electrode is exposed to air. Sepiolite possesses abundant hydrophilic groups that absorb water and drive it upward through capillary force. When water contacts sepiolite, the EDL will be formed. Under a certain pressure gradient, a relative displacement between solid and liquid will occur, resulting in a dynamic change in the EDL. Owing to the negative zeta potential, cations (e.g., H^+^) are more readily attracted and transported upward as water moves through the nanochannels of the sepiolite membrane during evaporation. Positive charges are primarily localized on the upper electrode, whereas negative charges are more densely distributed on the lower electrode. Upon connecting the S-WEG to an external circuit, a current signal coherent with the water flow direction is established. Conversely, the aluminum electrode repels negatively charged ions (OH^−^) in the aqueous medium, diminishing the H^+^ concentration at the bottom electrode. The surplus OH^−^ subsequently interacts with sepiolite, promoting ion generation at the bottom electrode and amplifying the concentration gradient between the bottom and top electrodes [41]. Notably, the aluminum electrode exhibits no signs of chemical corrosion following prolonged testing (Figure S5), demonstrating the device’s long-term operational stability.

### 3.3. Practical Applications of the S-WEG

**Figure 4:**
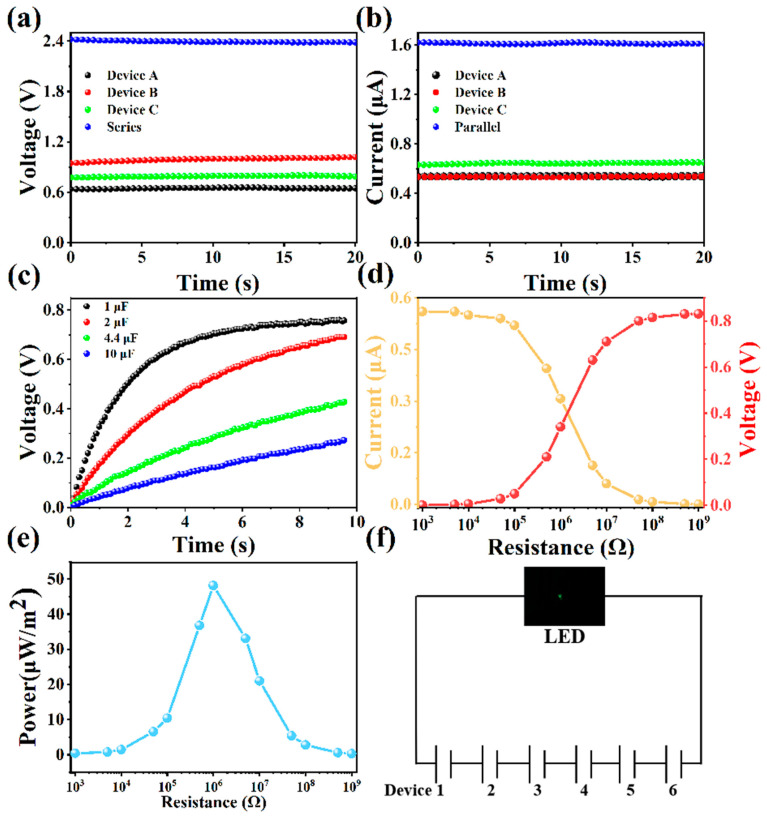
(**a**,**b**) The voltage and current generated by a single device/three devices connected in parallel and series, respectively. (**c**) Charging curves of an S-WEG for capacitors with different capacitances. (**d**) The output voltage and current of an S-WEG as a function of loading resistance. (**e**) Power density as a function of loading resistance. (**f**) Integrated S-WEG devices providing power for an LED light.

By connecting multiple S-WEGs, the output voltage and current can be well adjusted. As shown in Figure 4a,b, the voltage generated by three devices connected in series is 2.4 V, which is three times the output of a single S-WEG. The parallel test current of three devices can further amplify the current performance. As shown in Figure 4c, the capacitors of 1, 2, 4.4, and 10 μF can be charged to 0.75, 0.65, 0.38, and 0.23 V, respectively, within 8 s using a single S-WEG. Figure 4d shows the output current and voltage of an S-WEG as a function of external load resistance. When the load resistance is incrementally raised from 1000 Ω to 10 MΩ, the open-circuit voltage exhibits a monotonic increase from 0 V to 0.83 V, whereas the short-circuit current shows a corresponding decline from 0.56 μA to 0 μA. Figure 4e reveals that the peak output power density of 48.2 μW/m^2^ is achieved at a load resistance of 1 MΩ. Furthermore, Figure 4f highlights that a series connection of six S-WEG units enables direct operation of a commercial LED without capacitive energy storage. These results demonstrate that the S-WEG harnesses energy from water evaporation via self-powered electricity generation and delivers an immediate power supply to miniaturized electronics. In addition, as shown in Table S1, the nanogenerator based on sepiolite exhibits better overall performance than carbon black, carbon slurry, titanium dioxide nanoparticle film, and polymer material systems. The voltage can reach 0.9 V, and the current can reach 0.6 μA.

**Figure 5:**
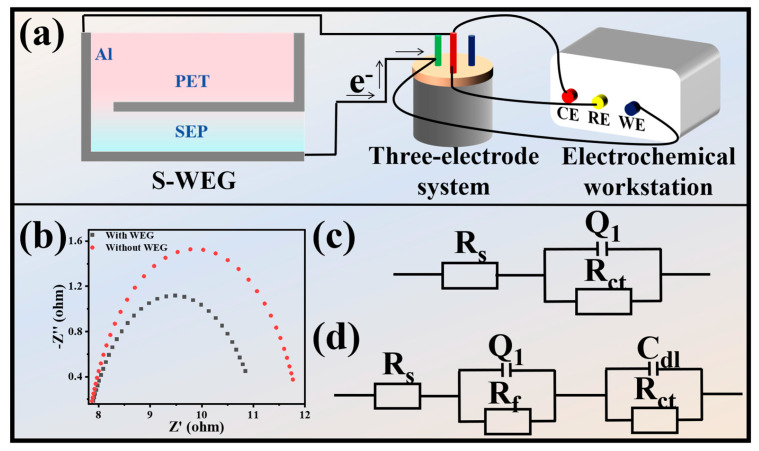
(**a**) Schematic diagram of an S-WEG protective iron sheet. (**b**) Nyquist plots of iron sheets with and without cathodic protection, powered by an S-WEG. Equivalent circuits of iron sheets connected (**c**) without and (**d**) with an S-WEG.

The S-WEG can be applied to cathodic protection. As shown in Figure 5a, the S-WEG is used to protect an iron sheet with dimensions of 2 cm × 1.6 cm. A three-electrode system was prepared using an iron electrode, a silver chloride electrode, and a platinum electrode as the working electrode, reference electrode, and counter electrode, respectively. The iron sheet electrode was submerged in a 3.5 wt% NaCl aqueous solution to emulate marine conditions. The S-WEG, capable of generating direct current, was integrated such that its anode and cathode were interfaced with the platinum and iron electrodes, respectively. Under these conditions, electrons migrate to the iron electrode surface, thereby establishing an efficient cathodic protection mechanism.

Figure 5b displays the electrochemical impedance spectroscopy data for the iron specimen. Notably, the Nyquist plot of the S-WEG-integrated iron electrode exhibits a reduced arc radius compared to the unprotected system, signifying diminished interfacial charge transfer resistance. The equivalent circuit diagram in Figure 5c,d incorporates *R*_s_ (electrolyte resistance), *Q*_1_ (constant phase element, CPE), and *R*_ct_ (charge transfer resistance). The incorporation of the S-WEG reduces the *R*_ct_ value of the iron substrate, where a lower *R*_ct_ corresponds to accelerated electron transfer kinetics. This reduction is attributed to the S-WEG’s output-driven polarization, which suppresses charge recombination. Collectively, these results validate the robust functionality of the S-WEG-enabled cathodic protection mechanism.

## 4. Conclusions

In this study, an S-WEG based on the natural mineral sepiolite was successfully developed. The generator utilizes the unique nanochannel structure and hydrophilicity of sepiolite to effectively convert water evaporation energy in the environment into electrical energy. By optimizing the evaporation height and width, the S-WEG achieves a short-circuit current of up to 0.5 μA and an open-circuit voltage output of 0.9 V. The experimental results show that the electrical output signal of the S-WEG is mainly driven by water evaporation caused by ion difference, rather than chemical battery effect. The S-WEG was also proved to be used for cathodic protection, protecting the metal surface from corrosion by directly outputting direct current and demonstrating its application prospects in the field of anti-corrosion.

## Data Availability

Data are available on request.

## Supplementary Materials

The following supporting information can be downloaded at https://www.mdpi.com/article/10.3390/nano15130983/s1: Figure S1. Crystal structure diagram of sepiolite. Figure S2. Voltage output of an S-WEG in a beaker sealed/unsealed with a cling film. Figure S3. The output voltage as a function of time when the S-WEG is covered by an acrylic box. Figure S4. Long-term stability test of the S-WEG. Figure S5. SEM image of the bottom Al electrode before and after a 40,000 s long-term test. Table S1. Comparison of energy harvesting performance between the sepiolite and previously reported water evaporation-based materials [42,43,44,45].
