# Supplementary material for: Sepiolite-Based Nanogenerator Driven by Water Evaporation
Source: Nanomaterials (Basel). 2025 Jun 25;15(13):983. doi: 10.3390/nano15130983 (PMC12251164; doi:10.3390/nano15130983)
Supplement: Supplementary file 1 [file nanomaterials-15-00983-s001.zip › nanomaterials-3684897-supplementary.pdf]

Table of contents

Figure S1. Crystal structure diagram of sepiolite
Figure S2. Voltage output when the generator is covering or uncovering with a box.
Figure S3. The change trend of voltage with time as the humidity rises.
Figure S4. SEM image of bottom Al electrode before and after 40000 s long-term test.
Figure S5. Long-term stability test of S-WEG.
Table S1. Comparison of previously reported materials for harvesting energy from water evaporation

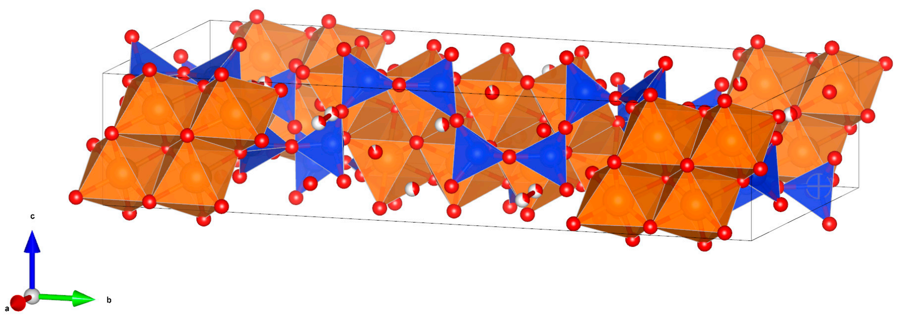

Figure S1. Crystal structure diagram of sepiolite.

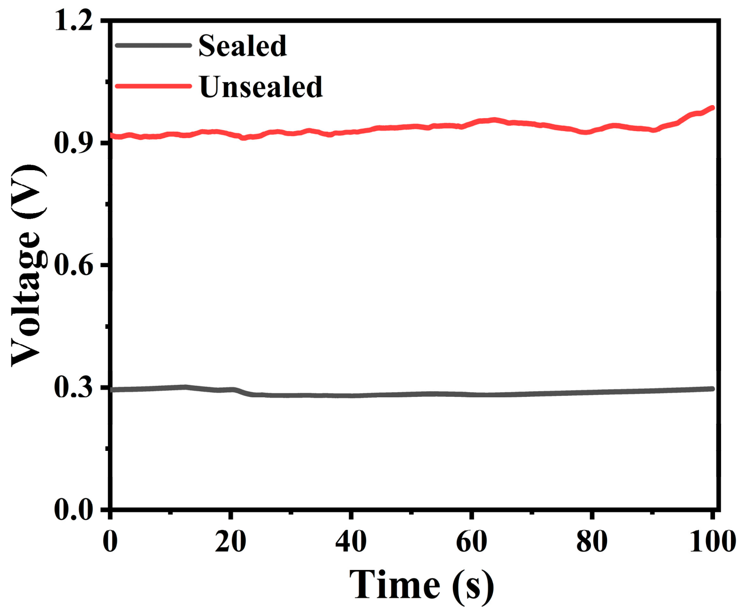

Figure S2. Voltage output of an S-WEG in a beaker sealed/unsealed with a cling film.

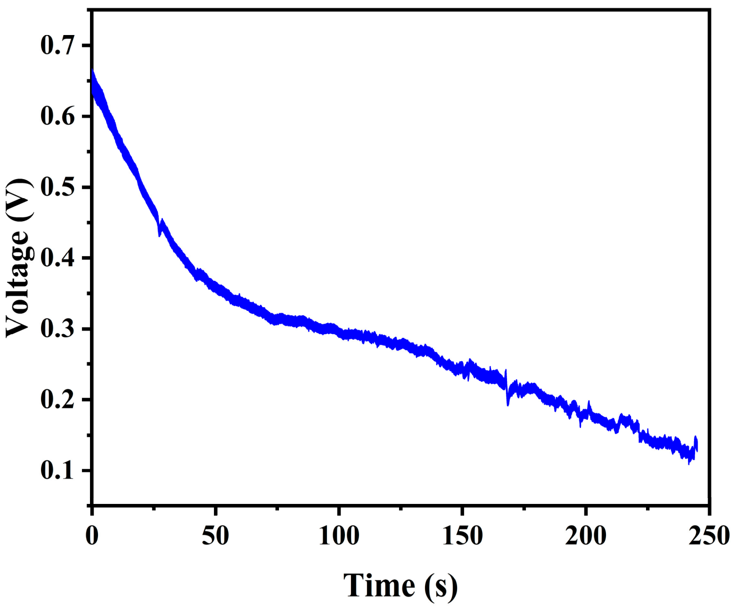

Figure S3. The output voltage as function of time as an S-WEG is covered by an acrylic box.

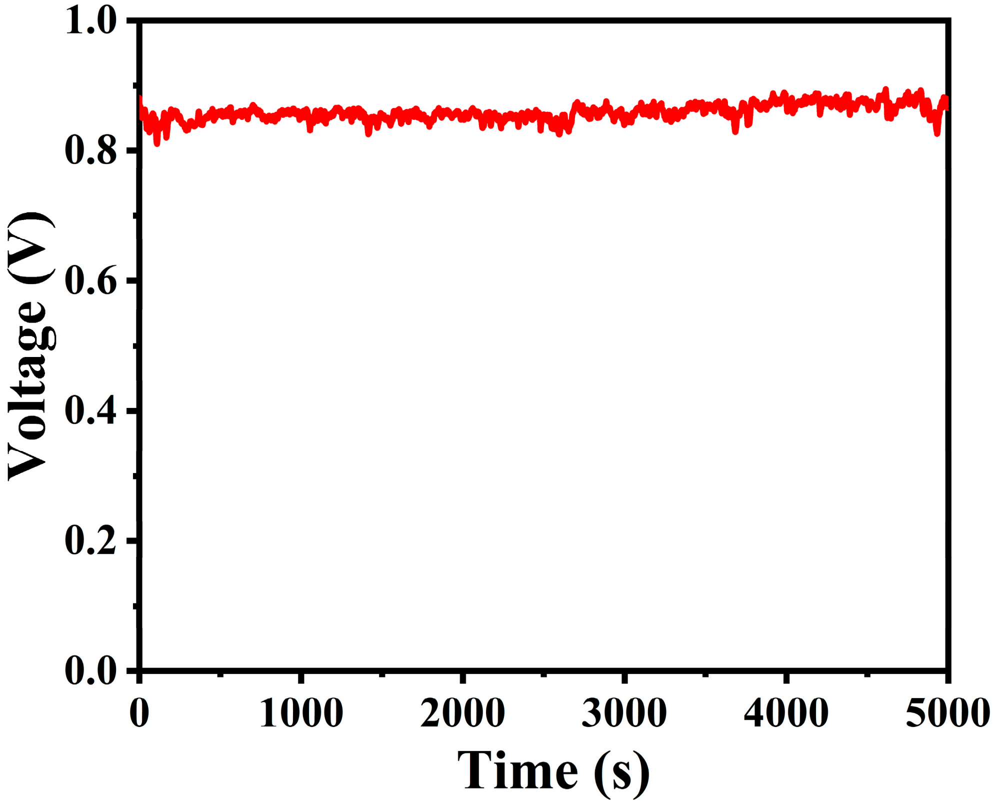

Figure S4. Long-term stability test of S-WEG.

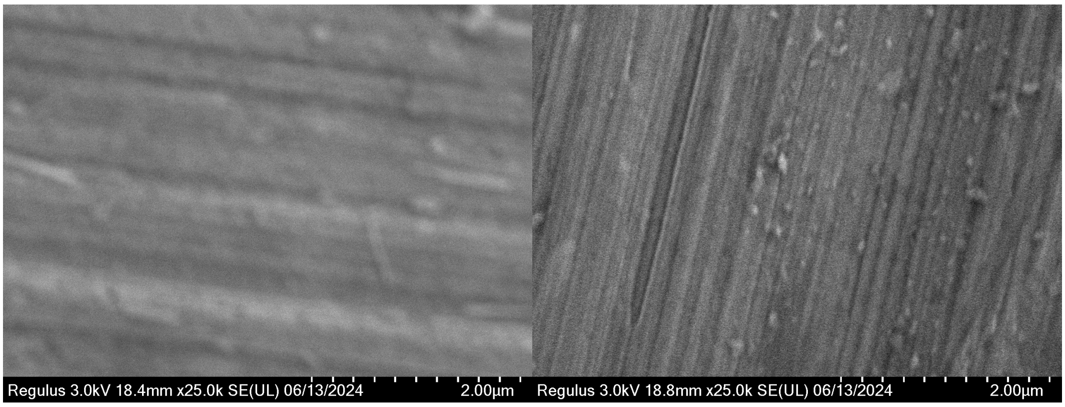

Figure S5. SEM image of bottom Al electrode before and after 40000 s long-term test.

Table S1. Comparison of energy harvesting performance between the sepiolite and previously reported water evaporation-based materials.

Materials	Voc (V)	Isc (μA)	Merit	Defect	Reference
Carbon black	0.79	~0.12	surface charging properties	high-resistance	[41]
Carbon slurry	1.2	0.5	surface charging properties	high-resistance	[42]
TiO_2 nanoparticle film	0.2	0.12	super-hydrophilicity	Easy to fall off	[43]
CBAP/PVDF	0.2	0.5	high flexibility	Low conductivity	[44]
Sepiolite	0.9	0.6	High hydrophilicity and unique nanochannels	Easy to fall off	This work
